# Investigation of correlates of protection against pharyngeal carriage of *Neisseria meningitidis* genogroups W and Y in the African meningitis belt

**DOI:** 10.1371/journal.pone.0182575

**Published:** 2017-08-10

**Authors:** Laura V. Cooper, Rahamatou Moustapha Boukary, Abraham Aseffa, Wude Mihret, Jean-Marc Collard, Doumagoum Daugla, Abraham Hodgson, Cheikh Sokhna, Babatunji Omotara, Samba Sow, Stephen Laryea Quaye, Kanny Diallo, Olivier Manigart, Martin C. J. Maiden, Helen Findlow, Ray Borrow, James M. Stuart, Brian M. Greenwood, Caroline L. Trotter

**Affiliations:** 1 Department of Veterinary Medicine, University of Cambridge, Cambridge, United Kingdom; 2 Centre de Recherche Médicale et Sanitaire (CERMES), Niamey, Niger; 3 Armauer Hansen Research Institute, Addis Ababa, Ethiopia; 4 Centre de Support en Santé International (CSSI), N'Djamena, Chad; 5 Navrongo Health Research Centre, Navrongo, Ghana; 6 Institut de Recherche pour le Développement, Dakar, Senegal; 7 Department of Community Medicine, University of Maiduguri, Maiduguri, Nigeria; 8 Centre pour les Vaccins en Développement, Bamako, Mali; 9 Faculty of Infectious Disease, London School of Hygiene & Tropical Medicine, London, United Kingdom; 10 Department of Zoology, University of Oxford, Oxford, United Kingdom; 11 Public Health England Vaccine Evaluation Unit, Manchester, United Kingdom; Universidad Nacional de la Plata, ARGENTINA

## Abstract

**Background:**

Serum bactericidal antibody titres that correlate with protection against invasive meningococcal disease have been characterised. However, titres that are associated with protection against acquisition of pharyngeal carriage of *Neisseria meningitidis* are not known.

**Methods:**

Sera were obtained from the members of a household in seven countries of the African meningitis belt in which a pharyngeal carrier of *N*. *meningitidis* had been identified during a cross-sectional survey. Serum bactericidal antibody titres at baseline were compared between individuals in the household of the carrier who became a carrier of a meningococcus of the same genogroup during six months of subsequent follow-up and household members who did not become a carrier of a meningococcus of this genogroup during this period.

**Results:**

Serum bacterial antibody titres were significantly higher in carriers of a serogroup W or Y meningococcus at the time of recruitment than in those who were not a carrier of *N*. *meningitidis* of the same genogroup. Serum bactericidal antibody titres to a strain of *N*. *meningitis* of the same genogroup as the index cases were no different in individuals who acquired carriage with a meningococcus of the same genogroup as the index case than in those who did not become a carrier during six months of follow-up.

**Conclusion:**

Serum bacterial antibody titres to *N*. *meningitidis* of genogroup W or Y in the range of those acquired by natural exposure to meningococci of these genogroups, or with cross-reactive bacteria, are not associated with protection against acquisition of carriage with meningococci of either of these genogroups.

## Introduction

Infection with *Neisseria meningitidis* (the meningococcus) usually results in an asymptomatic, or only mildly symptomatic, infection of the pharynx; invasion of the blood stream and invasive disease is a rare event. Study of pharyngeal carriage is, therefore, essential for a full understanding of the epidemiology of meningococcal infection [1]. The importance of carriage has been emphasised by the demonstration that the success of meningococcal conjugate vaccines in containing outbreaks of meningococcal disease in both industrialised countries [2,3] and in the African meningitis belt [4,5] is very dependent upon their ability to prevent carriage.

Studies with polysaccharide and conjugate vaccines have shown a strong correlation between vaccine induced serum bactericidal antibody (SBA) titre and protection against invasive meningococcal disease [6–8], an association that has been considered sufficiently robust to allow the licensure of meningococcal conjugate vaccines on the basis of immunogenicity and safety, without the requirement for a phase 3 efficacy trial [9,10]. However, little is known about the relationship between SBA and protection against pharyngeal carriage. A high SBA titre is associated with current carriage [11,12], with an increase in titre observed shortly following colonisation [13,14] and persistence for many months after clearance. A mathematical model confirmed that the relationship between the observed prevalence of group B meningococcal SBA and carriage by age of group B meningococci in the United Kingdom is consistent with a mechanism whereby episodes of carriage induce SBA activity [15]. In a study undertaken in Burkina Faso, both residence in an epidemic district and current carriage of group W *N*. *meningitidis* were independent predictors of high SBA titres, supporting the view that elevated titres may persist after clearance [16]. Taken together, this evidence suggests that SBA develop shortly after colonisation and remains elevated until clearance, after which SBA wane. However, whether SBA can prevent the acquisition of carriage of *N*. *meningitidis* and, if this is the case, how high a titre of antibody is required to achieve protection, is not known. Studies of pneumococcal and *Haemophilus influenzae* type b (Hib) infection suggest that the antibody titre required to prevent acquisition of carriage is higher than that needed to prevent invasive disease [17–20] but whether this is also the case for meningococcal disease has not been determined.

Members of the MenAfriCar Consortium have recently undertaken a series of studies of pharyngeal carriage of *N*. *meningitidis* in the African meningitis belt [21–23], including a study of patterns of transmission of the meningococcus within the household of a carrier identified during an initial cross-sectional survey [22]. A blood sample was obtained from each household member on entry into this study, allowing the relationship between the titre of bactericidal antibody present at the time of enrolment to the risk of becoming a carrier within the subsequent six months of follow-up to be investigated. The results of this investigation are described in this paper.

## Methods

### Field studies

Twenty cross-sectional surveys of meningococcal carriage were undertaken in seven countries across the African meningitis belt (Chad, Ethiopia, Ghana, Mali, Niger, Nigeria and Senegal) in 2010, 2011 and 2012 [22]. Households were selected randomly from an existing Demographic Surveillance System or from a census that was performed specifically for the study. Following provision of informed consent, up to five individuals per household, stratified by age (less than 1 year, 1–4 years, 5–14 years, 15–29 years and greater than 30 years) were recruited and a nasopharyngeal swab was taken to identify meningococcal carriers. Within four weeks of the identification of a carrier, all members of the carrier’s household were invited to participate in a longitudinal study [23]. Initial recruitment of a household to the longitudinal study was based on the detection of a putative carrier of *N*. *meningitidis* employing conventional microbiological methods, including serogrouping by agglutination. However, confirmatory molecular assays conducted at the University of Oxford resulted in some isolates being reclassified and this study is based on the results obtained following molecular characterisation of species and genogroup. A blood sample and a pharyngeal swab which had touched both the posterior pharynx and the tonsils were collected from all consenting members of the participating household at the initial visit. Swabs were obtained subsequently twice a month for two months and then monthly for a further four months up to a maximum of nine swabs per person [21]. Compliance was high and 88% of individuals provided at least six swabs. Transmission within households of genogroup A or C meningococci was detected only rarely and therefore SBA assays were undertaken only for genogroups W, X and Y. Exploration of the relationship between initial SBA titre and subsequent acquisition of carriage of genogroup W or Y meningococci was undertaken in samples obtained from households in Ethiopia, Ghana and Niger where transmission within households of meningococci belonging to these genogroups was detected relatively frequently.

### Laboratory methods

#### Isolation of *N*. *meningitidis*

The methods used to isolate and characterise *N*. *meningitidis* have been described previously [21]. In brief, pharyngeal swabs were plated directly onto Modified Thayer Martin agar plates in the field and taken to the laboratory within six hours of collection where they were incubated for 24–48 hours at 37°C in 5% CO_2_. Suspected *N*. *meningitidis* colonies identified on Thayer Martin plates were sub-cultured onto blood agar plates and tested using an oxidase test, followed by a Gram stain. All oxidase positive, Gram-negative diplococci underwent biochemical testing and those identified as *N*. *meningitidis* were serogrouped by slide-agglutination using antisera for serogroups A, W, X and Y. To confirm the identity of *Neisseria* identified by culture techniques, aliquots of boiled suspensions of all Gram-negative oxidase positive diplococci were sent to the University of Oxford for molecular analysis as described previously. Amplification and sequencing of a fragment of the *rplF* gene [24] was used to confirm the presence of, and differentiate between, *Neisseria* species [25]. Confirmed *N*. *meningitidis* were characterised further using a multiplex real time PCR assay detecting genogroups A, B, C, W, X and Y [26] and a sequencing assay detecting non-capsulated meningococci [27].

#### Serum bactericidal antibody assay

SBAs were carried out as described previously [28]. Baby rabbit serum (Pel-Freeze) was used as the exogenous source of complement. Bactericidal antibody titres were expressed as the reciprocal of the final serum dilution giving 50% killing after 60 minutes incubation. For computational purposes, titres <4 were assigned a value of 2. Samples from Niger were tested against *N*. *meningitidis* serogroup W reference strain, W:P1.18–1,3 (M01 240070). Samples from Ghana from households with at least one genogroup W carrier were tested against two group W strains: a reference strain, W:P1.18–1,3 (M01 240070) and a local strain, W:P1.5,2. Samples from Ethiopia were tested against two test strains: Y:P1.5,2 (M00242975) and X:P1.5–1,10–1.

### Statistical methods

The terms used in the data analysis and their definitions are summarised in Table 1. Index carriers were defined as individuals within the household who were carriers of a meningococcus of the SBA test genogroup at visit 0 (the initial cross-sectional survey) or visit 1. For individuals with differing isolates at visits 0 and 1, determinate results were favoured over non-determinate (ND) results. Only carriage events of the same genogroup as the index strain were considered. Index carriers were defined as individuals carrying a meningococcus of the SBA test genogroup at visit 0 or 1. Determination of SBA titre in subjects who were a carrier on visit 1 and those who were not, allowed for an examination of the correlation between current carriage and SBA. Current carriers were stratified as those carrying a strain of the same genogroup and the same *porA* variable regions 1 and 2 as the SBA test strain, those carrying a strain with the same genogroup but different *porA*, those carrying a strain with a different genogroup but the same *porA*, and those carrying a strain with a different genogroup and *porA*. Secondary carriers were defined as non-index carriers who acquired a meningococcus of the SBA test strain genogroup at least once during the study period. Non-carriers were defined as non-index carriers who did not acquire a meningococcus belonging to the test strain genogroup at any time during the six-month follow-up period.

**Table 1:** Index and subsequent carriers by genogroup.

Genogroup	Index carriers (Number)	Subsequent carriers (Number)
A	0	0
W	45	28 [28]
X	1	0
Y	13	7 [7]
Non-groupable	4	1 [1]

Genogroup of the index case carrier and the number of additional carriers of *N*. *meningitidis* of the same genogroup detected within these households in Ethiopia, Ghana or Niger during six months of follow-up.

Logistic regression was used to identify risk factors for *Nm*W acquisition, in particular serologic status. Variables that attained univariate significance at P < 0.1 were added to a multivariable model that included age group, serological status, and sex *a priori*. Those with a P value of < .05 were retained. As a final check, dropped variables were re-entered into the model one at a time; the variable was retained if P < .05.

Geometric mean titres (GMTs) and 95% confidence intervals were calculated using standard methods. Two different SBA reciprocal titres were evaluated as hypothetical correlates of protection against acquisition of pharyngeal carriage of *N*. *meningitidis*. A reciprocal tire of 8 was selected based on the correlate of protection against invasive serogroup C meningococcal disease [29], consistency with studies that have evaluated the immunogenicity of quadrivalent meningococcal conjugate vaccines [30,31] and the titre used in other seroprevalence studies [32]. A reciprocal titre 128 was selected as an alternative cut-off based on the distribution of titres found among participants in the present study. Because no carriage of any meningococci with the *porA* P1.18,3 family (homologous with the group W reference strain) was detected in the study, serological correlates of protection against acquisition of such strains could not be evaluated.

### Ethics

The purpose and methods of the study were explained to community leaders at community meetings and through the media. Written, informed consent for obtaining a pharyngeal swab and a blood sample was obtained from adults and for the children under their care. Written informed assent was also obtained from participants aged 12 years or more. Oral assent was obtained from younger children. Consent and assent forms were translated into the relevant local language.

The study protocols, consent and assent forms were approved by the LSHTM Ethics Committee and by the ethics committees of each of the African partner institutions with the exception of Chad, which does not have a formal ethical committee, and where approval for the activities of the consortium was granted by a committee set up to oversee MenAfriCar studies by the Ministry of Health (S1 Table).

## Results

### Study participants

Nine hundred and ninety-three individuals from 147 households were recruited to the study, 747 (75%) of whom consented to have a blood sample taken. Two hundred and twenty-three samples were obtained from individuals resident in households in which a carrier of genogroup W or Y was detected. One hundred and ninety were tested by SBA assay against one strain of meningococcus of the corresponding genogroup and 33 against multiple strains. The mean age of the individuals tested was 19.5 years, 51% were female. Fifty-one percent reported receiving MenAfriVac or a “meningitis vaccine” during the time of a PsA-TT mass vaccination campaign.

### Index carriers and spread of *N*. *meningitidis* within household

The characteristics of the index carriers in the households recruited to the household study and the subsequent transmission of meningococci of the same genogroup in these households has been described in detail previously [23]. The genogroup distribution of the index carrier in the households recruited to this serological study are summarised in Table 1. Because of the infrequent occurrence of carriage of genogroup A *N*. *meningitidis* among the index cases and the small number of genogroup X transmission events recorded, evaluation of serological correlates of protection against acquisition of carriage with *N*. *meningitidis* has been restricted to meningococci of genogroup W or Y.

### Distribution of SBA titres and relation to acquisition of carriage

**Fig 1:**
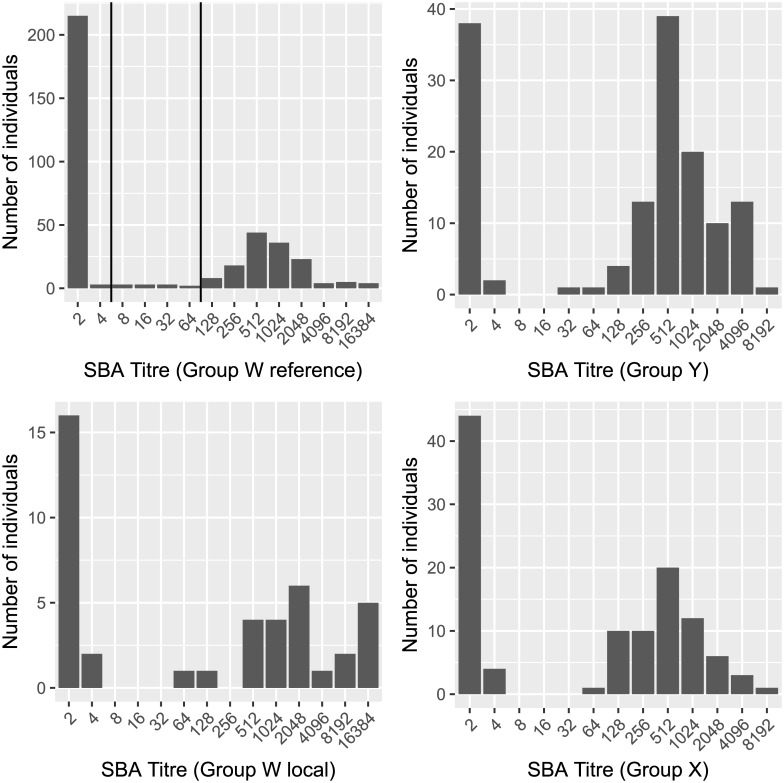
SBA titre distribution. Overall distribution of SBA titres in all household members tested against the reference strains of *N*. *meningitidis* genogroups W, X and Y and against a local Ghanaian genogroup W strain.

The overall distribution of SBA titres was bimodal for genogroups W, X and Y with a significant proportion of “non-responders”–individuals with a titre of less than 8 –and an approximately normal distribution of ‘‘responders” with titres around a median of 512 (Fig 1). The numbers of individuals with a titre of 8 or greater and those with a titre of 128 or greater among index carriers, secondary carriers, and those who never carried are shown in (Table 2). Individuals with a titre of genogroup W SBA of 128 or greater of were found significantly more frequently among current carriers than among individuals in the other two groups (p < 0.01 and p < 0.01 respectively).

**Table 2:** Antibody titres by exposure and outcome.

	Serum bactericidal antibody titre		
	<8 No. [%]	≥8 No. [%]	≥ 128 No. [%]
Genogroup W			
Carrier on recruitment	9 [20]	36 [80]	35 [78]
Not a carrier on recruitment	209 [64]	117 [36]	107 [33]
Subsequent carrier	37 [62]	23 [38]	21 [35]
In households with a carrier	18 [64]	10 [36]	9 [32]
Non-carrier	172 [65]	94 [35]	86 [32]
In households with a carrier	67 [59]	46 [41]	43 [38]
Genogroup Y			
Carrier on recruitment	1 [9]	10 [91]	9 [82]
Not a carrier on recruitment	39 [30]	92 [70]	91 [69]
Subsequent carrier	1 [14]	6 [86]	6 [86]
In households with a carrier	1 [14]	6 [86]	6 [86]
Non-carrier	38 [31]	86 [69]	85 [69]
In households with a carrier	6 [32]	13 [68]	12 [63]

The number and percentage of individuals with an initial SBA of 8 or greater, or with a titre of 128 or greater in initial carriers, subjects who subsequently became a carrier of *N*. *meningitidis* of the same genogroup as the index case and those who did not become a carrier of *N*. *meningitidis* of this genogroup during the six months of follow-up.

**Fig 2:**
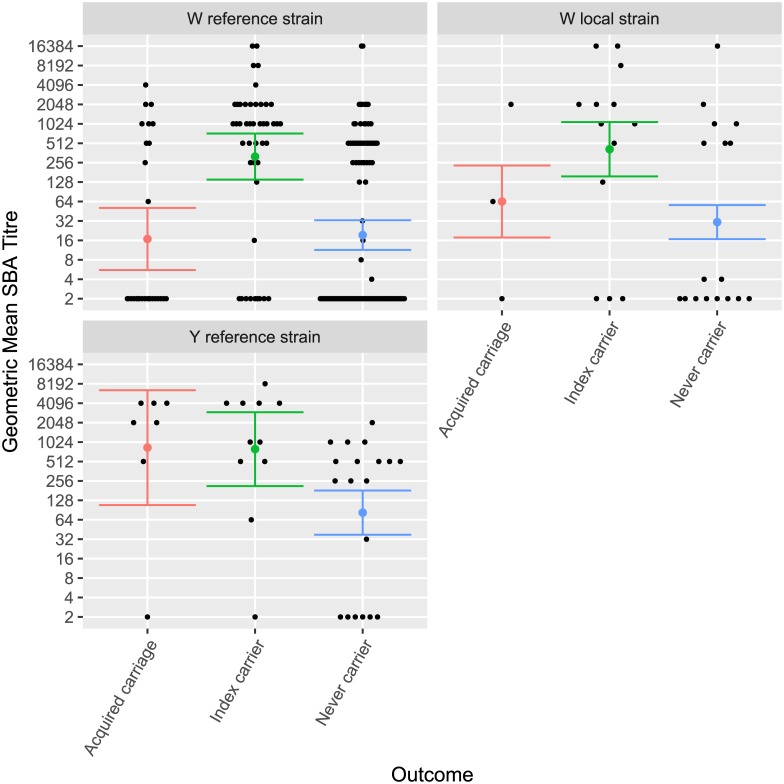
Geometric mean by exposure and outcome. Geometric mean SBA titres and 95% CI found in current carriers of *N*. *meningitidis* genogroup W or Y and in household members exposed to a carrier who became a carrier of *N*. *meningitidis* of the same genogroup as the index case and among those who did not.

**Fig 3:**
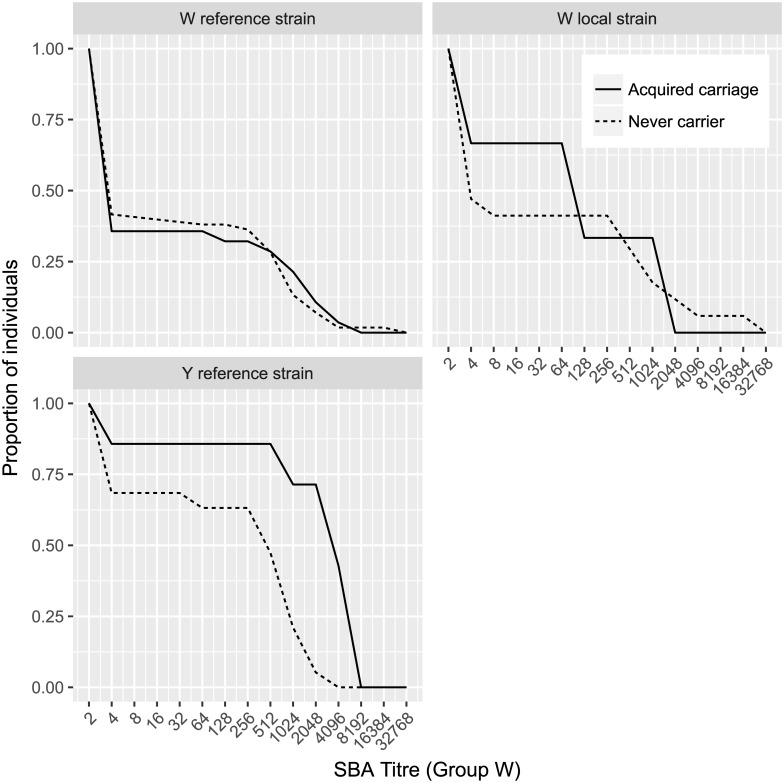
Reciprocal titre by exposure and outcome. Reciprocal plots of SBA titre found in individuals exposed to a carrier of *N*. *meningitidis* who became a carrier of a meningococcus belonging to the same genogroup as the index carrier during six months of follow-up and those who did not. Sera tested against a genogroup W reference strain, genogroup W local strain, genogroup Y reference strain.

Geometric mean titres were significantly higher among index carriers for the group W reference strain (360 [95% CI 190–660]) compared with the GMT in the other two groups (21 [95% CI 12–36] and 14 [95% CI 12–16] respectively) (Fig 2). There was no difference in GMT against the Ghana W strain and the group W reference strain between index carriers and secondary carriers, although both were significantly higher than non-carriers. The distribution of titres at the time of recruitment between individuals who were not carrying at the time of recruitment but who became a carrier of a meningococcus of the same genogroup as index case and those who did not is shown in Fig 3. The patterns are very similar with no suggestion that titres on enrolment were higher in individuals who did not become a carrier than in those who did.

### Risk factors for genogroup W acquisition

Risk factors for acquisition of a genogroup W strain at the individual level are shown in Table 3. Age, meningococcal vaccination status and crowding were associated with higher odds of acquisition of carriage in a univariate analysis but only age remained a significant risk factor in a multivariate logistic analysis. Having a group W SBA titre of less than 8 was not a significant risk factor for acquisition, suggesting that SBA titres greater than or equal to 8 cannot be considered a correlate of protection against carriage acquisition. The same results were achieved with a higher cut-off threshold of 128.

**Table 3:** Factors influencing odds of carriage acquisition.

Factor	Subjects (Secondary carriers)	Crude OR (95% CI)	Adjusted OR (95% CI)
Group W SBA titre			
<8	165 (29)	1.0	1.0
≥8	103 (21)	1.20 (0.64–2.24)	1.13 (0.59–2.15)
Age (years)			
<5	55 (13)	2.61 (0.98–7.47)	2.60 (0.98–7.46)
5–14	70 (21)	3.61 (1.48–9.82)	3.50 (1.42–9.59)
15–29	66 (7)	1.0	1.0
≥30	77 (9)	1.12 (0.39–3.30)	1.12 (0.39–3.31)
Sex			
Female	143 (24)	1.0	1.0
Male	125 (26)	1.30 (0.70–2.42)	1.14 (0.60–2.17)
Household exposure			
No group W index carrier	141 (26)	1.0	…
Group W index carrier	127 (24)	1.03 (0.55–1.91)	…
Vaccination status			
Not vaccinated	84 (10)	1.0	…
Any history of vaccination	184 (40)	2.06 (1.01–4.56)	…
Crowding			
<4 people per bedroom	92 (10)	1.0	…
≥4 people per bedroom	176 (40)	2.41 (1.19–5.34)	…

Univariable and multivariable logistic regression analysis of factors associated with genogroup W *N*. *meningitidis* acquisition.

### Influence of the test strain on SBA geometric mean titre

SBA GMTs to the serogroup W reference strain (W:P1.18–1,3) were significantly higher among carriers of genogroup W strains than among carriers of heterologous strains (320 [95% CI 140–720] versus 7.3 [95% CI 4.0–13]). This was not true for serogroup Y SBA GMTs, and could not be evaluated for serogroup X or the Ghanaian serogroup W strain. Titres against the local Ghanaian group W strain and the group W reference strain were highly correlated (Pearson’s rho of 0.95, n = 42). For the 12 individuals carrying the Ghana strain at the time of recruitment, titres against this strain were tightly correlated with titres against the reference W strain (Pearson’s rho = 0.99, p < 0.001)

## Discussion

We have investigated the role of serum bactericidal antibodies in acquisition of meningococcal carriage by following households with known exposure from an index carrier. Surprisingly, we found no evidence of an association between SBA titre and protection against acquisition of group W or Y carriage in subjects exposed to a meningococcus of the homologous genogroup. This was the case even after correction for other variables such as age, sex, and place of residence. Serum bactericidal antibodies, acquired as a result of natural exposure or vaccination, are associated with protection against invasive meningococcal disease [6–9] but we are not aware of any previous study which has investigated the association of SBA with protection against acquisition of meningococcal carriage. Previous studies of subjects vaccinated with Hib or pneumococcal conjugate vaccines have suggested that higher titres of IgG antibody are required to protect against pharyngeal carriage than against invasive disease [17–19], as might be expected if protection is dependent upon diffusion of antibodies from the blood to the mucosa.

There are a number of possible explanations for our finding of a lack of correlation between SBA titres and risk of acquisition of pharyngeal carriage with *N*. *meningitidis*. It is possible that SBAs were performed using a strain which was not able to detect protective antibodies against the strains circulating in the study households. However, we found a very close correlation between responses to the W reference strain compared to the locally isolated genogroup W strain. In addition, SBA titres of individuals who were a carrier on entry into the study had a significantly higher SBA titre than individuals who were not a carrier suggesting that the assay was detecting responses to the local strain making strain variation an unlikely explanation for our findings. Secondly, it is possible that natural exposure to infection does not induce SBA at a high enough titre to provide protection whilst vaccination may do so. Evidence to support this hypothesis is provided by the fact that the GMT of SBA to *N*. *meningitidis* genogroup W or Y found in individuals in this study (< 1000) was lower than the GMT found in adolescents or young adults one month after vaccination with a quadrivalent meningococcal conjugate vaccine employing a SBA that used baby rabbit serum as a source of complement (8390 [95% CI 7777, 9051] for genogroup W and 13865 [95% CI 12968, 114824] for genogroup Y respectively [33]. Similarly, the GMT induced by the serogroup A conjugate vaccine MenAfriVac was 4700 (95% CI 4300, 5100) one month after vaccination in those aged 2 to 29 years and 6200 (95% CI 4900, 7900) in those aged 1 to 2 years [9]. However, some individuals in this study with titres of 4096 and higher, within the range of responses induced by these conjugate vaccines, became a carrier of a meningococcus of the same genogroup. Thirdly, it is possible that the affinity or isotype of antibodies induced by natural infection is different from that induced by conjugate vaccination and less able to provide protection against acquisition of carriage. Fourthly, it is possible that in the harsh environment of the African meningitis belt up-regulation of CECAM expression facilitates binding of meningococci to the epithelium even in the presence of moderately high concentrations of anti-capsular antibody. In other words, meningococci may carry the gene for a particular capsule but may or may not express it under different circumstances, and this means that an immune response targeting primarily the capsular antigen may not protect against future acquisition. Finally, it is possible that the primary means of naturally acquired protection against carriage of the meningococcus is not mediated though serum bactericidal antibody but by locally induced mucosal immune mechanism or by a cell mediated immune response as has been suggested to be the case for *S*. *pneumoniae* [34], a possibility that warrants further investigation.

This study has a number of weaknesses. Firstly, it was not possible to test all sera collected during the household studies against all genogroups and some selection of samples was required. This was done on the basis of selecting samples which would be able to provide the strongest evidence of whether or not SBAs were protective against acquisition of carriage. Subjects were selected on the basis of known exposure to a carrier and from households where transmission events with genogroup W or Y meningococci dominated. Secondly, we have assumed that subjects who became carriers and those did not were equally exposed to the index carrier and this may not have been the case, although the crowded nature of most of the study households make this likely. Age is likely to have been an important factor influencing contact with the carrier and the lack of association between SBA and protection against acquisition of carriage persisted even after an age correction had been made. Age will also have influenced likely number of previous infections. All study sites fall within the meningitis belt, and as such experience frequent epidemics. In particular, outbreaks (greater than 60 suspected cases per 100,000 population annually) were documented in 2006 in both sites in Niger and in 2010–2012 in both sites in Ghana. [35]

This study has shown that the SBA titres induced by natural exposure to infection with *N*. *meningitidis* of genogroup W or Y are not associated with protection against acquisition of carriage of meningococci of the same genogroup, as might have been expected. A more detailed study which provided more information on patterns of contact within a household and which studied immunological correlates in addition to SBA could be rewarding.

## Supporting information

S1 Table Ethical review panels. Ethical review panels giving approval to the study in each African centre.
(PDF)
