# Supplementary material for: Investigation of correlates of protection against pharyngeal carriage of Neisseria meningitidis genogroups W and Y in the African meningitis belt
Source: PLoS One. 2017 Aug 10;12(8):e0182575. doi: 10.1371/journal.pone.0182575 (PMC5552120; doi:10.1371/journal.pone.0182575)
Supplement: S1 Table — Ethical review panels giving approval to the study in each African centre. (PDF) [file pone.0182575.s001.pdf]

Table S1. Ethical review panels. Ethical review panels giving approval to the study in each African centre.

Country	Ethical review board
Ethiopia	AHRI-ALERT Ethics Review Committee
Ghana	Navrongo Health Research Centre Institutional Review Board
Mali	Ethics Committee of the Faculty of Medicine, University of Bamako
Niger	National Ethics Committee of Niger
Nigeria	Research and Ethics Committee of the University of Maiduguri Teaching Hospital
Senegal	National Ethics Committee for Health Research
Chad	Special committee of the Ministry of Health
